# Solitary Fibrous Tumor of the Parotid Gland: A Case Report

**Published:** 2015-09

**Authors:** Ryan Yu, Ryan Rebello

**Affiliations:** 1 *Department of Pathology and Molecular Medicine, McMaster University, Hamilton, Ontario, Canada.*; 2 *Department of Radiology, McMaster University, St. Joseph’s Hospital, Hamilton, Ontario, Canada.*

**Keywords:** Parotid gland, Parotid diseases, Solitary fibrous tumors

## Abstract

**Introduction:**

Solitary fibrous tumor is a rare, mesenchymal neoplasm that has been reported in numerous sites. Occurrence in the parotid gland is exceedingly rare.

**Case Report:**

A 53-year-old man with a 2 cm solitary fibrous tumor of the left parotid gland, that was observed clinically and operatively and thought to be a neoplasm arising from Stensen's duct, is described. A pre-operative CT scan demonstrated a well-circumscribed, solid, avidly-enhancing nodule superficial to the masseter muscle, deep to the platysma, and intimately associated with the parotid duct. Multiple fine needle aspirations yielded scant fibrous tissue and lymphocytes. A superficial parotidectomy was performed. The histopathological and immunohistochemical findings were in keeping with solitary fibrous tumor, fibrous variant, with a low mitotic rate and a peripherally-entrapped parotid duct surrounded by abundant periductal collagen and lymphocytes. At a 2-year follow up, there was no evidence of tumor recurrence or metastasis.

**Conclusion:**

Solitary fibrous tumor should be suspected in the context of a slow-growing, well-circumscribed, solid, avidly-enhancing nodule of the parotid gland. Grossly intimate association with the parotid duct may reflect peripheral entrapment. Fine needle aspirations that predominantly yield collagen without spindle cell clusters should be correlated with clinical and radiological findings, as it is expected in tumor sampling of the fibrous variant. Although solitary fibrous tumor of the parotid gland usually exhibits benign behavior, it is best regarded as potentially malignant. Patient management and follow-up should be tailored to each individual and clinicopathological risk assessment of the recurrent/metastatic potential.

## Introduction

Solitary fibrous tumor (SFT) is a rare mesenchymal neoplasm. It was recognized as a distinctive entity in 1931 by Klemperer and Rabin, who reported it as a primary neoplasm of the pleura (1). Since then, SFT has been reported in numerous sites, including the lung, pericardium, abdomen, pelvis, breast, adrenal gland, genitourinary tract, soft tissue, periosteum, and head and neck. Occurrence in the parotid gland is exceedingly rare, which makes preoperative diagnosis challenging. We describe a patient with SFT of the parotid gland, that clinically and operatively mimicked a tumor arising from Stensen's duct. 

## Case Report

**Fig1:**
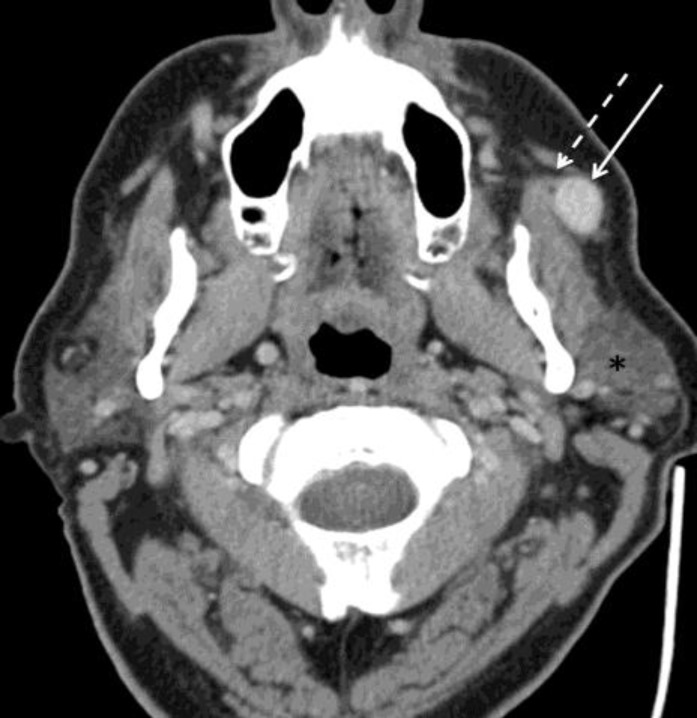
Axial contrast-enhanced CT image demonstrates a solid avidly enhancing nodule (white solid arrow) superficial to the masseter muscle and deep to the platysma. It is intimately associated with the parotid duct (dashed white arrow). The mass is anterior to the parotid gland (*).

A 53-year-old man presented to a head and neck surgeon with a slow-growing nodule at the anterior aspect of the left Stensen's duct. His medical history indicated that he suffered from chronic obstructive pulmonary disease and a remote dental abscess. Computed tomography (CT) scan of the neck showed a circumscribed, enhancing oval mass along the course of the left parotid duct immediately superficial to the left masseter muscle (Fig. 1). 

There was no cervical lymph node enlargement. Fine needle aspiration (FNA), performed 3 times, yielded scant fibrous tissue and lymphocytes. He was taken to the operating room for local resection with a preoperative diagnosis of a tumor arising from the Stensen's parotid duct. The lesion was isolated, confirmed as contiguous with the duct, and removed together. 

Frozen section was interpreted as a spindle cell tumor. Sacrifice of the parotid duct was necessary and a superficial parotidectomy was performed. Two small buccal branches of the facial nerve were divided in the course of the resection and repaired. Postoperative facial nerve function was normal. At a 2-year follow-up, there was no evidence of tumor recurrence or metastasis.

The lesion was a firm, well-circumscribed, homogeneous pink-tan mass measuring 2 cm x 1.7 cm x 1 cm. Upon microscopic examination, the tumor was composed of spindle cells arranged in haphazard short interlacing fascicles. 

**Fig 2:**
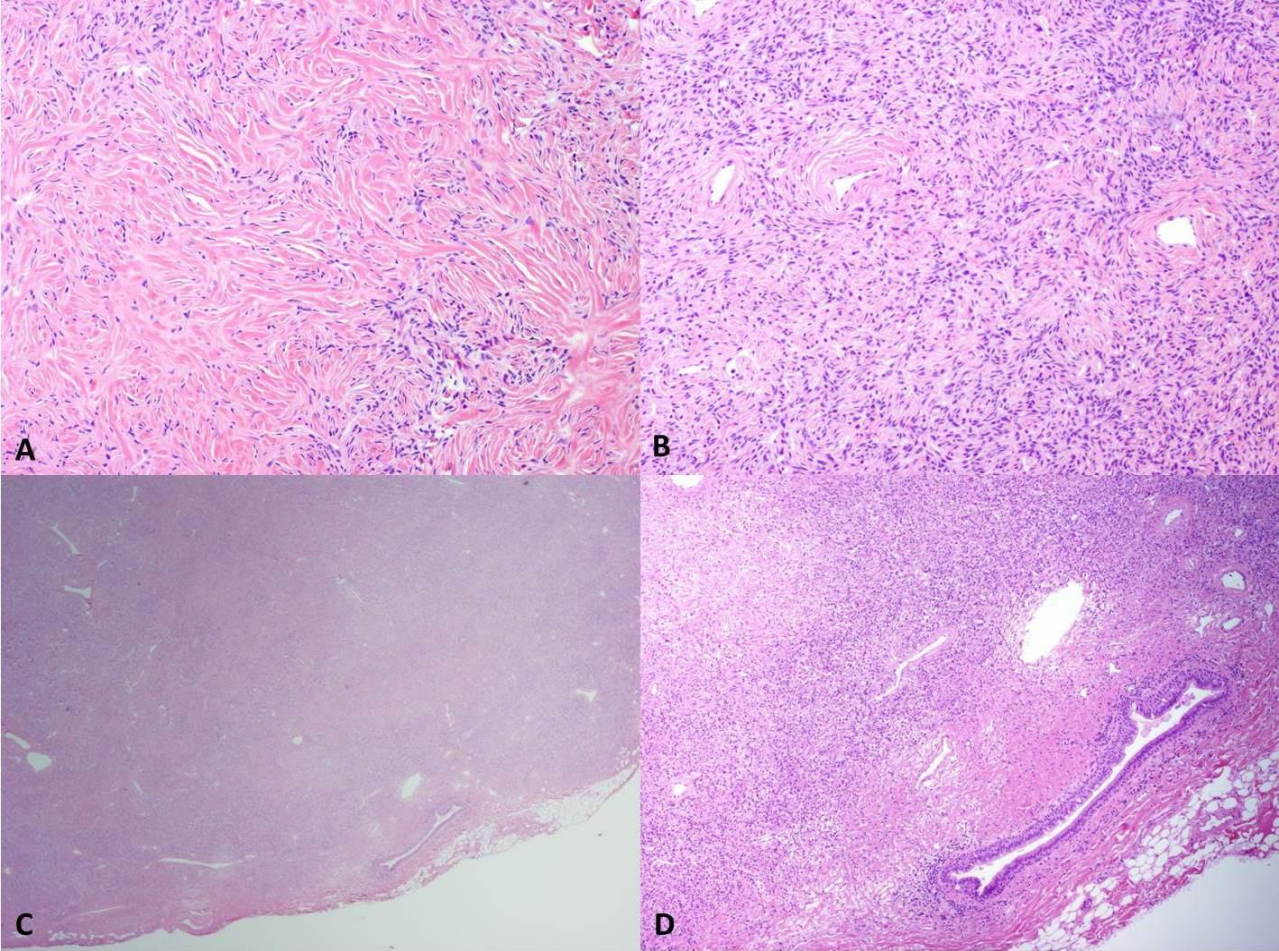
**A**) The tumor is composed of bland round-to-spindle cells separated by keloid-like collagen (H&E, 100x).  **B**) Blood vessels with thickened, hyalinized walls (H&E, 100x). **C**) The tumor is well-circumscribed with the parotid duct entrapped at the periphery, accounting for the clinical and operative impression of a tumor arising from the parotid duct (H&E, 12.5x). **D**) Parotid duct with abundant periductal collagen and lymphocytes (H&E, 40x).

The tumor cells had tapering, bland-looking nuclei, and were separated by keloid-like collagen (Fig. 2A).

Mitoses were identified at less than 1 per 10 high-power fields (HPF). 

Necrosis was absent. Medium-sized branching vessels within the tumor showed thickened and hyalinized walls (Fig.2B). Stensen’s duct was identified, entrapped at the periphery of the tumor (Fig.2C), with abundant periductal collagen and lymphocytes (Fig. 2D). The tumor cells showed diffusely positive immunostaining for CD34, CD99, and Bcl-2; focally positive staining for CD68; and no immunoreactivity for pan-cytokeratin, EMA, α-SMA, h-caldesmon, desmin, S100 protein, CD31, p63, and ALK-1. The gross, histopathological, and immunohisto chemical findings were in keeping with a solitary fibrous tumor. 

## Discussion

SFT of the parotid gland is very rare. There are 26 previously reported cases in English literature (Medline 1946-2014, Embase 1974-2014). Together with the current case, the patients include 15 males and 12 females, ranging in age from 11 to 79-years-old (average 50.7 years, median 47 years). Only one patient was pediatric and diagnosed concurrently with type 1 neurofibromatosis (2). SFT of the parotid gland presents itself as a painless, firm, slow-growing lesion of a few months to few years in duration. Symptoms of obstructive sleep apnea are relatively common and may be related to parapharyngeal extension of the tumor. Facial nerve palsy is rare. Upon CT examination, SFTs appear as solitary, well-defined masses that are hypointense to muscle and demonstrate heterogeneous contrast enhancement. On magnetic resonance imaging, SFTs are homogeneous and isointense to muscle. On T1- and T2-weighted images; they are heterogeneous and mildly hyperintense. They are also strongly enhanced post-gadolinium in areas of mild hyperintensity on T2-weighted images (3). 

SFT of the parotid gland is a smooth-contoured mass that ranges in size from 1 to 18 cm (average 4.8 cm, median 4 cm) with a white cut surface. Histologically, parotid SFTs reported to date, including this current case, are of the conventional or “fibrous variant” as proposed by Gengler and Guillou (4). The tumors have a pushing border and are composed of round-to-spindle cells with bland or vesicular nuclei in a variably collagenous stroma. In areas of high cellularity, the tumor cells adopt fascicular, herringbone, or storiform architecture. In areas of low cellularity, thick collagen bundles separate the tumor cells. Myxoid stromal change may be found. Numerous mid-caliber vessels are scattered throughout the tumor and may be elongated or branched with thickened, hyalinized walls. Scattered multinucleated giant cells or a mild lymphoplasmacytic infiltrate may be found. Focal entrapment of normal serous acini or small ducts at the edge of the tumor is not uncommon. However, the current case uniquely illustrates the peripheral entrapment of Stensen’s duct, which accounts for the clinical and operative impression of a tumor arising from Stensen’s duct.

Pre-operative diagnosis of parotid SFT by FNA is challenging, largely because its rarity renders little to no diagnostic suspicion. Previous cases in which FNA was performed have been interpreted as pleomorphic adenoma, myoepithelial tumor, and cementifying fibroma. Further, because the fibrous variant of SFT has alternating hypercellular and hypocellular fibrous areas, FNA interpretation is subject to sampling bias. Sampling of hypercellular areas yields sheets and three-dimensional clusters of monomorphic fusiform spindled cells in a background of capillaries and wispy collagen (5). However, sampling of hypocellular fibrous areas yields predominantly stromal collagen, which might be interpreted as non-diagnostic.

The immunohistochemistry of parotid SFT recapitulates that of SFT at other sites. The tumor cells typically show positive staining for vimentin, CD34, Bcl-2, and CD99, and negative staining for cytokeratin, EMA, CAM5.2, SMA, desmin, GFAP, CD117, and S100 protein. In combination with morphology, the immunoprofile is supportive in distinguishing SFT from a range of mimics, including nodular fasciitis, myoepithelioma, spindle cell carcinoma, spindle cell lipoma, spindle cell melanoma, schwannoma, desmoplastic fibroblastoma, dermatofibrosarcoma protuberans, and malignant peripheral nerve sheath tumor. Further, nuclear expression of STAT6 has been found to be a highly sensitive immunohistochemical marker for SFT (6) and may be useful in small biopsies, CD34-negative SFTs, and distinguishing SFTs from other mesenchymal tumors with significant immunohistochemical overlap.

Complete resection is the cornerstone of management. Short follow-up periods (0.1 to 5.6 years) have been performed and most reported patients have no evidence of disease at follow-up. However, one patient had pulmonary metastases and two patients had a local recurrence (7-9). 

Predicting the clinical behavior of SFT remains a challenging clinicopathological problem. Demicco et al. (10) found that patient age (≥55years), tumor size (>15 cm), and mitotic activity (≥4 per 10 HPF) predict the development of SFT metastasis and have devised a risk stratification model that may be clinically useful. 

## Conclusions

Solitary fibrous tumor should be suspected in the clinical context of a slow-growing, well-circumscribed, solid, avidly-enhancing nodule of the parotid gland. Intimate association with the parotid duct may reflect peripheral entrapment. Fine needle aspirations that predominantly yield collagen without spindle cell clusters should be correlated with clinical and radiological findings.

Although solitary fibrous tumor of the parotid gland usually exhibits benign behavior, it is best regarded as potentially malignant. Patient management and follow-up should be tailored to the clinicopathologic risk of the recurrent/ metastatic potential. 

## References

[1] Klemperer P, Rabin CB (1931). Primary neoplasms of the pleura. Arch Pathol.

[2] Thompson M, Cheng LH, Stewart J, Marker A, Adlam DM (2004). A paediatric case of a solitary fibrous tumour of the parotid gland. Int J Pediatr Otorhinolaryngol.

[3] Liu Y, Tao X, Shi H, Li K (2014). MRI findings of solitary fibrous tumours in the head and neck region. Dentomaxillofac Radiol..

[4] Gengler C, Guillou L (2006). Solitary fibrous tumour and haemangiopericytoma: evolution of a concept. Histopathology.

[5] Bauer JL, Miklos AZ, Thompson LD (2012). Parotid gland solitary fibrous tumor: a case report and clinicopathologic review of 22 cases from the literature. Head Neck Pathol.

[6] Doyle LA, Vivero M, Fletcher CD, Mertens F, Hornick JL (2014). Nuclear expression of STAT6 distinguishes solitary fibrous tumor from histologic mimics. Mod Pathol.

[7] Messa-Botero OA, Romero-Rojas AE, Chinchilla Olaya SI, Díaz-Pérez JA, Tapias-Vargas LF (2011). Primary malignant solitary fibrous tumor/ hemangiopericytoma of the parotid gland. Acta Otorrinolaringol Esp.

[8] Suárez Roa Mde L, Ruíz Godoy Rivera LM, Meneses García A, Granados-García M, Mosqueda Taylor A (2004). Solitary fibrous tumor of the parotid region Report of a case and review of the literature. Med Oral.

[9] Alonso-Rodríguez E, González-Otero T, Castro-Calvo A, Ruiz-Bravo E, Burgueño M (2014). Parotid gland solitary fibrous tumor with mandibular bone destruction and aggressive behavior. J Clin Exp Dent.

[10] Demicco EG, Park MS, Araujo DM, Fox PS, Bassett RL, Pollock RE (2012). Solitary fibrous tumor: a clinicopathological study of 110 cases and proposed risk assessment model. Mod Pathol.

